# Phylogenetic reconstruction of the genus *Purpureocillium* based on multilocus nuclear and mitochondrial DNA sequences, with the description of *Purpureocillium xinfangense* sp. nov. (Ophiocordycipitaceae, Hypocreales) from Southwestern China

**DOI:** 10.3389/ffunb.2026.1972355

**Published:** 2026-10-13

**Authors:** Xiaoyan Liu, Hao Wang, Lijun Luo, Zuoheng Liu, Yangtao Yu, Baomin Fan, Hong Yu

**Affiliations:** 1 School of Mathematics and Computer Science, Yunnan Minzu University, Kunming, Yunnan, China; 2 The International Joint Research Center for Sustainable Utilization of Cordyceps Bioresources in China and Southeast Asia, Yunnan University, Kunming, Yunnan, China; 3 Yunnan Herbal Laboratory, School of Chemistry and Environment, Yunnan Minzu University, Kunming, Yunnan, China

**Keywords:** mitochondrial phylogeny, multigene phylogeny, *Purpureocillium*, species, taxonomy

## Abstract

**Introduction:**

The phylogenetic relationships within the genus Purpureocillium remain incompletely resolved. In this study, we reconstructed the phylogeny of Purpureocillium using multilocus nuclear and mitochondrial sequence data and evaluated the taxonomic status of a newly collected fungal species from southwestern China.

**Methods:**

Phylogenetic analyses were conducted using five nuclear loci (*ITS, 18S, 28S, TEF1-α,* and *RPB1*) and mitochondrial gene sequences. Morphological characteristics were examined for the newly collected fungus and compared with those of *P. lilacinum*, *P. lavendulum*, and *P. takamizusanense*. Mitochondrial genomes of these four species were also analyzed for genome structure, gene composition, and nucleotide characteristics.

**Results:**

Morphological and phylogenetic evidence supported the recognition of a new species, *Purpureocillium xinfangense*. It was characterized by conidiophores bearing two or three whorls of phialides, predominantly short cylindrical phialides with a mucronate apex, and elongated conidia arranged in chains. Mitochondrial phylogenetic analysis placed *P. xinfangense* in a distinct lineage with high topological stability. The mitochondrial genomes of all four examined species were circular, whereas the genome of *P. xinfangense* was approximately four to five times larger than those of the other three species. All four species showed positive GC skew values. Among the 15 protein-coding genes, nad5 was the longest and *atp8* the shortest, while tRNA genes ranged from 71 to 87 bp.

**Discussion:**

Although *P. xinfangense* is currently known from a single collection, its distinctive morphology, rare ant-associated substrate, and concordant support from nuclear and mitochondrial phylogenetic datasets justify its recognition as a distinct evolutionary lineage. These findings expand current knowledge of species diversity and mitochondrial genome evolution within *Purpureocillium*.

## Introduction

Luangsa-Ard et al. (2011) established a novel fungal genus, *Purpureocillium*, within the phylum Ascomycota, class Sordariomycetes, order Hypocreales, and family Ophiocordycipitaceae, based on comparative analyses of the 18S rRNA gene (*18S*), internal transcribed spacer *(ITS*), and partial translation elongation factor 1-α (*TEF1-α*) sequences in relation to *Paecilomyces lilacinus*. *Purpureocillium* is a widely distributed genus of fungi found in diverse terrestrial and marine ecosystems. It has been frequently isolated from various substrates such as soil, insects, nematodes, and root nodules of multiple crop species (Rédou et al., 2015; Srilakshmi et al., 2017; Liu et al., 2019; Chen et al., 2020a; Pedezzi et al., 2021). Notably, certain species within this genus are recognized as opportunistic pathogens that can cause severe infections in humans, with ocular infections being the most commonly reported, followed by cutaneous and subcutaneous manifestations (Liu et al., 2019; Chen et al., 2020b; Jagadeesh et al., 2022).

*Purpureocillium lilacinum* Luangsa-ard, Hywel-Jones, Houbraken & Samson and *P. lavendulum* Perdomo, Dania García, Gené, Cano & Guarro are broad-spectrum biocontrol fungi that demonstrate significant efficacy against a diverse array of plant pests and pathogens (Luangsa-Ard et al., 2011; Perdomo et al., 2013). These fungal species exhibit effective control over plant-parasitic nematodes, particularly *Meloidogyne incognita* (Kofoid & White) Chitwood, as well as other prominent pests such as *Trialeurodes vaporariorum* (Westwood) Quaintance & Baker, *Frankliniella occidentalis* (Pergande) Karny, *Aphis gossypii* Glover, *Tetranychus urticae* Koch, and *Acromyrmex lundii* (Guérin-Méneville) Forel. Furthermore, they display inhibitory activity against various plant pathogens, including Botrytis spp., bacterial agents, and even viral pathogens (Castillo Lopez et al., 2014; Xie et al., 2016; Jagadeesh et al., 2022). The biological control mechanisms of *P. lilacinum* have been thoroughly investigated, revealing its capacity to secrete chitinase and serine protease enzymes. These enzymes degrade the epidermal structures of nematodes, leading to premature egg hatching or parasitic mortality (Park et al., 2004; Sharma et al., 2014).

Early mitochondrial genomic studies of the genus *Purpureocillium* have reported complete mitogenomes for *P. lilacinum* and *P. lavendulum* (Li et al., 2019; Liang et al., 2021). Subsequently, Chang et al. (2024) performed a comparative mitogenomic analysis across four *Purpureocillium* species—*P. lilacinum*, *P. lavendulum*, *P. takamizusanense*, and *P. atypicola*—revealing substantial interspecific variation in mitochondrial genome size and intron content, whereas the core mitochondrial gene order was largely conserved. Notably, their analysis demonstrated that intron gain and loss events constitute the primary driver of genome size divergence within the genus. This comparative framework serves as a critical reference for interpreting the markedly expanded mitochondrial genome of *P. xinfangense* identified in the present study.

In this study, we integrated morphological and molecular phylogenetic evidence to reconstruct the phylogenetic relationships within the genus *Purpureocillium*. A comprehensive dataset was employed, consisting of five nuclear gene fragments (*ITS*, *18S*, *28S*, *TEF1-α*, and *RPB1*) and 14 protein-coding mitochondrial genes. Phylogenetic analyses were conducted using both the maximum likelihood (ML) and the Bayesian inference (BI) approaches. A novel fungal species, *P. xinfangense*, was discovered and formally described based on specimens collected in Yunnan Province, China. Morphological comparisons among *P. lilacinum*, *P. lavendulum*, *P. takamizusanense*, and *P. xinfangense* were provided using expository text rather than formal taxonomic descriptions for the previously described taxa. The mitochondrial genomes of these four species were sequenced using BGI sequencing technology to investigate their gene composition, genomic structure, and GC content. This study contributes to a deeper understanding of species diversity and distribution within the genus *Purpureocillium* and provides essential taxonomic insights for future systematic and ecological research. The new species is currently represented by a single gathering; however, the combination of its rare ant-associated substrate, distinctive cylindrical conidia, and congruent support from nuclear and mitochondrial phylogenies provides the basis for its formal description.

## Materials and methods

### Sample collection and isolation

*Purpureocillium xinfangense* strains were isolated from an ant-associated fungal substrate collected from Xinfang Reservoir in Nanping Town, Pu′er City, Yunnan Province, China (100°57′43″E, 22°42′19″N; altitude: 1380 m). The infected ant cadaver was surface-sterilized by immersion in 30% hydrogen peroxide for 5–8 min, followed by immersion in 75% ethanol for 1 min, and subsequently rinsed five times with sterile distilled water. After drying on sterile filter paper, the cadaver was divided into four segments (head and abdomen each comprising one segment), inoculated onto antibiotic-supplemented PDA medium, and incubated at 25 °C. Neither the ant host nor the associated *Ophiocordyceps unilateralis* species complex was formally identified in this study; therefore, species-level taxonomic assignments are not provided for either the host or the fungal pathogen. Voucher specimens have been deposited at the Yunnan Herbarium (YHH), and the corresponding cultures are preserved at the Yunnan Fungal Culture Collection Center (YFCC).

### Morphological studies

Cultures from PDA plates were transferred to malt extract agar (MEA) plates and incubated at 25 °C for 7–14 d. After incubation, colony diameters were measured. For morphological analysis, microscope slides were prepared by transferring mycelial fragments from the cultures onto blocks of MEA medium (5 mm in diameter). Phialides, conidia, and conidiophores were observed using a synthetic dye solution. Micromorphological features were examined and measured using Olympus CX40 and BX53 microscopes (Olympus Corporation, Tokyo, Japan), following Wang et al. (2020).

### DNA extraction, PCR amplification, and sequencing

The isolates were cultured on MEA for 7–14 d at 25 °C. Total genomic DNA was extracted using the CTAB method, as described by Liu et al. (2001). Polymerase chain reactions (PCRs) were performed in a final volume of 50 µL containing 1× Taq PCR Master Mix (Tiangen, Beijing, China), 0.10 µM of each primer, and 1 ng template DNA. According to the manufacturer’s formulation of the 2× master mix, the final reaction contained 250 µM of each dNTP and 1.5 mM MgCl_2_. Sterile double-distilled water (ddH_2_O) was used for PCR preparations. The following primer pairs were used: NS1 and NS4 (White et al., 1990) for the *ITS* region; LR0R (Vilgalys and Hester, 1990) and LR5 (Rehner and Samuels, 1994) for the *28S* region; EF1α-EF and EF1α-ER (Bischoff et al., 2006; Sung et al., 2007) for *TEF1-α*; and RPB1/RPB1Cr_oph (Liu et al., 1999; Araújo et al., 2018) for *RPB1*. Gene amplification and sequencing were carried out in accordance with Wang et al. (2020).

### DNA sequence alignments and phylogenetic analysis

Phylogenetic analyses were conducted based on the concatenated sequences of five genes *(ITS*, *18S*, *28S*, *TEF1-α*, and *RPB1*). The gene sequences were obtained from the GenBank database and combined with those generated in this study. Detailed taxonomic information and GenBank accession numbers for these sequences are presented in **Table 1**. The concatenated nuclear-gene alignment used for phylogenetic analysis is provided as Data File 1. Sequence alignments were carried out using Clustal X version 2.0 and MEGA6 software (Larkin et al., 2007; Tamura et al., 2013). After alignment, the sequences were concatenated. Potential incongruences among datasets were assessed using PAUP* 4.0b10 (Swofford, 2002), which indicated no significant conflicts in phylogenetic signals across the five genes. Phylogenetic trees were constructed using Bayesian inference (BI) and maximum likelihood (ML). ModelFinder (Kalyaanamoorthy et al., 2017) implemented in PhyloSuite v1.2.3 was employed to select the optimal nucleotide substitution model based on the Bayesian information criterion (BIC). Bayesian inference analysis was performed with MrBayes version 3.1.2 (Ronquist and Huelsenbeck, 2003) under the JC+I+G+F model for 2 million generations. Maximum-likelihood analysis was carried out using RAxML version 7.0.3 (Stamatakis, 2006) under the TN+I+G4+F model with 5 000 bootstrap replicates. Accordingly, model selection was completed prior to the Bayesian inference and maximum-likelihood analyses.

**Table 1:** GenBank accession numbers of sequences used in this study.

Species	Voucher	ITS	18S	28S	*TEF1-α*	*RPB1*	Reference
*Drechmeria campanulata*	IMI 356051^T^		AF339592	AF339543			Sung et al. (2001)
*Drechmeria panacis*	CBS 142798^T^		MF588890	MF588897	MF614144		Yu et al. (2018)
*Drechmeria sphaerospora*	CBS 522.80^T^		AF339590	AF339541			Sung et al. (2001)
*Drechmeria zeospora*	CBS 335.80^T^		AF339589	AF339540	EF469062	EF469091	Sung et al. (2001)
*Harposporium helicoides*	ARSEF 5354		AF339577	AF339527			Sung et al. (2001)
*Harposporium harposporiferum*	ARSEF 5472^T^		AF339569	AF339519	DQ118747	DQ127238	Sung et al. (2001)
*Tolypocladium cylindrosporum*	ARSEF 2920			MH871712	MG228390	MG228384	Vu et al. (2019)
*Tolypocladium ophioglossoides*	CBS 100239		KJ878910	KJ878874	KJ878958	KJ878990	Quandt et al. (2014)
*Tolypocladium subparadoxum*	NBRC 106958	OP207727	OP207715	OP207735	OP223149	OP223127	Dong et al. (2022)
*Purpureocillium atypicola*	CEM 1185		KJ878907	KJ878872	KJ878955		Quandt et al. (2014)
*Purpureocillium atypicola*	OSC 151901		KJ878914	KJ878880	KJ878961	KJ878994	Quandt et al. (2014)
*Purpureocillium atypicola*	CBS 744.73	GU980041	EF468987	EF468841	EF468786	EF468892	Sung et al. (2007)
*Purpureocillium atypicola*	RCEF 3833		KJ878913	KJ878879	KJ878960		Quandt et al. (2014)
*Purpureocillium xinfangense*	**YFCC 22109449**	**PQ649434**			**PQ852093**		**This study**
*Purpureocillium lilacinum*	CBS 284.36		AY526475	FR775484	EF468792	EF468898	Sung et al. (2007)
*Purpureocillium lilacinum*	NHJ 3497		EU369096	EU369033	EU369014	EU369053	Johnson et al. (2009)
*Purpureocillium lilacinum*	FMR 8648	FR734089		FR775472	FR734144	FR775495	Perdomo et al. (2013)
*Purpureocillium lilacinum*	FMR 10380	FR734102		FR775485	FR734157	FR775508	Perdomo et al. (2013)
*Purpureocillium lilacinum*	**YFCC 22109448**	**PQ649433**	**PQ649622**	**PQ649625**	**PQ852092**	**PQ852089**	**This study**
*Purpureocillium takamizusanense*	NHJ 3582		EU369097	EU369034	EU369015		Johnson et al. (2009)
*Purpureocillium takamizusanense*	RCEF4811	MT568626	MW718227	MW718253	MT583722	MW723137	Ban et al. (2015)
*Purpureocillium takamizusanense*	NBRC 100742	LC008197			LC008333		Ban et al. (2015)
*Purpureocillium takamizusanense*	NBRC 110234	LC008207			LC008341		Ban et al. (2015)
*Purpureocillium takamizusanense*	**YFCC 22094450**		**PQ649621**	**PQ649624**	**PQ852091**	**PQ852088**	**This study**
*Purpureocillium lavendulum*	FMR 10452	FR734107		FR775490	FR775517	FR775513	Perdomo et al. (2013)
*Purpureocillium lavendulum*	FMR 10376	FR734106		FR775489	FR775516	FR775512	Perdomo et al. (2013)
*Purpureocillium sinense*	TR05091			PV082920.1			Chen et al. (2025)
*Purpureocillium atlanticum*	FLOR73173		PX571242.1	PX571243.1			Araújo et al. (2025)
*Purpureocillium hyophilae*	MFLUCC:24-0325			PQ453486.1			Gunarathne et al. (2025)
*Purpureocillium roseum*	IOM 325363.2			MT560198.1			Calvillo-Medina et al. (2021)
*Purpureocillium sodanum*	AH07D04			PP380701.1			GenBank
*Purpureocillium zongqii*	TK042			PQ211285.1			Chen et al. (2024b)
*Polycephalomyces formosus*	ARSEF 1424		KF049615	AY259544	DQ118754	DQ127245	Chaverri et al. (2005)
*Polycephalomyces paracuboideus*	NBRC 101742		KF049611	KF049630	KF049685	KF049647	Kepler et al. (2013)

Bold text indicates sequences generated in this study.

### Sequencing, assembly, and annotation of mitogenome

The extracted DNA was preserved in liquid nitrogen and then transported to Shenzhen Huada Gene Co. for sequencing by BGI. The initial annotation of the mitochondrial genome was conducted using MITOS (Galaxy; usegalaxy.org) with the RefSeq 89 fungi database. MFannot (https://megasun.bch.umontreal.ca/apps/mfannot/) was applied to predict mitochondrial genes based on a set of four defined patterns, referencing the published mitochondrial genomes of *P. lilacinum* NC056378 and *P. lavendulum* MW019427 from NCBI (https://www.ncbi.nlm.nih.gov/). The resulting annotations were cross-validated against these reference genomes to ensure accuracy. Mitochondrial genome maps for the four species were constructed using OGDRAW (Greiner et al., 2019). The AT content, GC content, and protein-coding genes (PCGs) of each species were analyzed using PhyloSuite v1.2.3 (Zhang et al., 2020). Sequence features of the mitochondrial genomes, including gene composition, number of PCGs, rRNA genes, tRNA genes, and other relevant characteristics, were analyzed using Geneious Prime v2023.2.1. For annotation tables, the distance between adjacent genes was calculated as (start of downstream gene – end of upstream gene – 1). Positive values indicate intergenic spacer length (non-coding distance between adjacent features), while negative values indicate the length of gene overlap. For overlapping genes, the absolute value of the negative number represents the overlap length. In the tables, intergenic spacer length is reported as a non-negative value, and any overlap between adjacent features is recorded separately in the designated overlap column, rather than represented as a negative intergenic spacer.

### Phylogenetic analyses of mitogenomes

Mitochondrial genome sequences of 52 samples representing six fungal families (Bionectriaceae, Clavicipitaceae, Cordycipitaceae, Hypocreaceae, Nectriaceae, and Ophiocordycipitaceae) were obtained from GenBank (**Table 2**). Fourteen mitochondrial protein-coding genes (PCGs)—*atp6*, *atp8*, *atp9*, *cob*, *cox1*, *cox2*, *cox3*, *nad1*, *nad2*, *nad3*, *nad4*, *nad4L*, *nad5*, and *nad6*—were concatenated for phylogenetic reconstruction. As for the nuclear dataset, best-fit substitution models were selected with ModelFinder (Kalyaanamoorthy et al., 2017) implemented in PhyloSuite v1.2.3 under the BIC, with JC+I+G+F used for BI and TN+I+G4+F used for ML. The concatenated mitochondrial protein-coding-gene alignment used for phylogenetic analysis is provided as Data File 2.

**Table 2:** Species information for the construction of the mitochondrial-genome phylogenetic tree of Hypocreales.

Family	Species	GenBank ID	Voucher
Bionectriaceae	*Acremonium chrysogenum*	NC023268	ATCC:11550
	*Acremonium fuci*	NC029851	3a34
	*Acremonium implicatum*	NC026534	–
	*Clonostachys compactiuscula*	MW030498	YFCC 897
	*Clonostachys rogersoniana*	MW030499	YFCC 899
	*Clonostachys rosea*	NC036667	6792
Clavicipitaceae	*Epichloe festucae*	NC032064	AR5
	*Epichloe typhina*	KX066185	E8
	*Metacordyceps chlamydosporia*	NC022835	170
	*Metarhizium anisopliae*	AY884128	ME1
	*Metarhizium robertsii*	JELW01000367	ARSEF 2575
	*Orbiocrella petchii*	MT447058	SD3
Cordycipitaceae	*Beauveria bassiana*	KT201149	e17
	*Beauveria caledonica*	KT201150	fhr1
	*Beauveria malawiensis*	KT201147	k89
	*Cordyceps brongniartii*	EU100743	IMBST95031
	*Cordyceps cicadae*	MH922223	CCAD02
	*Cordyceps militaris*	KP722505	F02
	*Cordyceps tenuipes*	MK234910	YFCC 2017002
	*Lecanicillium muscarium*	AF487277	–
	*Lecanicillium saksenae*	KT585676	–
	*Parengyodontium album*	KX061492	ATCC:56482
Hypocreaceae	*Hypomyces aurantius*	NC030206	–
	*Paecilomyces penicillatus*	NC043850	SAAS_ppe1
	*Trichoderma asperellum*	NC037075	B05
	*Trichoderma gamsii*	KU687109	KUC1747
	*Trichoderma hamatum*	MF287973	–
	*Trichoderma hamatum*	NC036144	–
	*Trichoderma reesei*	NC003388	QM9414
Nectriaceae	*Fusarium commune*	NC036106	JCM11502
	*Fusarium mangiferae*	NC029194	–
	*Fusarium oxysporum*	MF155191	mh2-2
	*Fusarium proliferatum*	LT841261	ITEM2400
	*Gibberella moniliformis*	NC016687	7600
	*Ilyonectria destructans*	NC030340	2007/P/476
	*Nectria cinnabarina*	NC030252	5175
Ophiocordycipitaceae	*Hirsutella rhossiliensis*	NC030164	USA-87-5
	*Ophiocordyceps sinensis*	NC034659	–
	*Purpureocillium lavendulum*	MW019427	YMF1.00683
	*Purpureocillium lilacinum*	NC056378	WS1608
	*Tolypocladium guangdongense*	NC054274	GD15
	*Tolypocladium cylindrosporum*	NC046839	ARSEF963
	*Tolypocladium inflatum*	KY924883	ARSEF 616
	*Tolypocladium ophioglossoides*	NC031384	L2
Outgroup	*Neurospora crassa*	KY498478	FGSC 9718
	*Penicillium citrinum*	NC047444	JH1205

## Results

### Phylogenetic analyses based on nuclear genes

The concatenated dataset included five loci: *ITS*, *18S*, *28S*, *TEF1-α*, and *RPB1*. Sequences from *Polycephalomyces* formosus Kobayasi and *Po. paracuboideus* (S. Ban, Sakane & Nakagiri) Kepler & Spatafora were used as outgroups. The phylogenetic tree (**Figure 1**) was inferred using both the maximum likelihood (ML) and the Bayesian Inference (BI) methods based on the combined *ITS*-*18S*-*28S-TEF1-α-RPB1* dataset. The resulting trees displayed congruent topologies within the genus *Purpureocillium* and related genera. Notably, *P. roseum* and *P. sodanum* formed a well-supported clade with *P. lilacinum* in the phylogenetic analysis, suggesting closer evolutionary relationships among these three taxa. However, given that sequences for *P. roseum* and *P. sodanum* in the NCBI database are currently limited to the *ITS* and *28S* regions, the *ITS* data showed phylogenetic incongruence with the other loci and resulted in unstable placement of these taxa in the combined analyses. Therefore, only the *28S* sequences were included in the present study, their phylogenetic placement should be interpreted cautiously and requires confirmation using additional loci. The fungal isolate obtained from Yunnan Province was consistently and strongly supported within the *Purpureocillium* clade.

**Figure 1:**
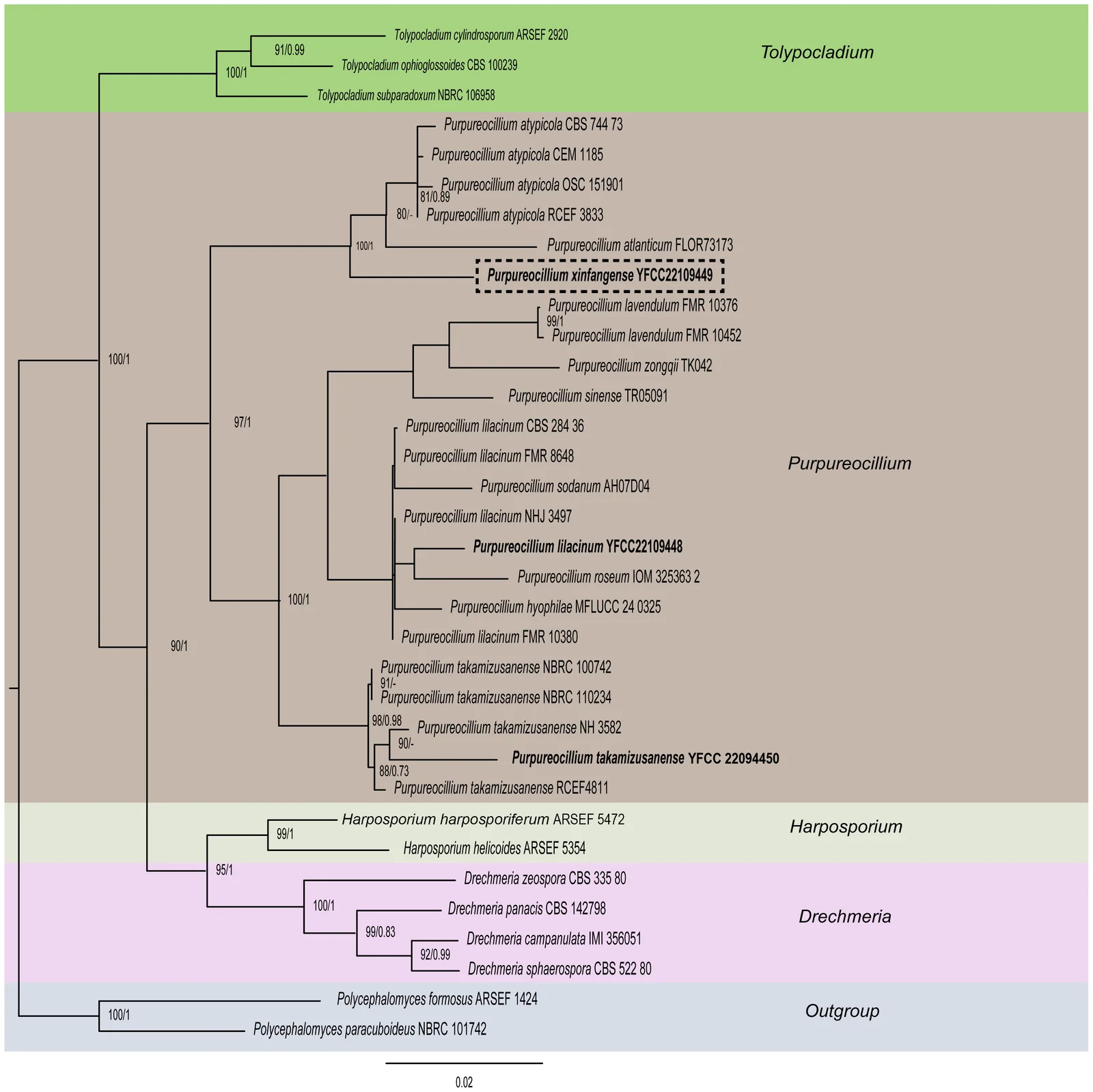
Phylogenetic tree reconstructed from a concatenated dataset of *ITS*, *18S*, *28S*, *TEF1-α*, and *RPB1* sequences using Bayesian inference (BI) and maximum likelihood (ML). Node support values are shown as Bayesian posterior probabilities (PP ≥ 0.70) and maximum-likelihood bootstrap support values (BS ≥ 70%). The scale bar (0.02) indicates the expected number of substitutions per site.

### Phylogenetic analysis based on mitochondrial genes

The phylogenetic tree was inferred from concatenated amino acid sequences of 14 mitochondrial protein-coding genes (PCGs) across the genus *Purpureocillium*. *Neurospora crassa* Shear & B.O. Dodge and *Penicillium citrinum* Thom were designated as outgroups. Phylogenetic analyses were conducted under both Bayesian inference (BI) and maximum likelihood (ML) frameworks, with nodal support assessed via BI posterior probabilities and ML bootstrap proportions (1,000 replicates). Within the order Hypocreales, six monophyletic families—Nectriaceae, Clavicipitaceae, Ophiocordycipitaceae, Hypocreaceae, Cordycipitaceae, and Bionectriaceae—were strongly supported. All four *Purpureocillium* species included in this study formed distinct, well-supported clades (**Figure 2**). Notably, *P. xinfangense* constituted a phylogenetically isolated lineage, sister to the remaining *Purpureocillium* taxa.

**Figure 2:**
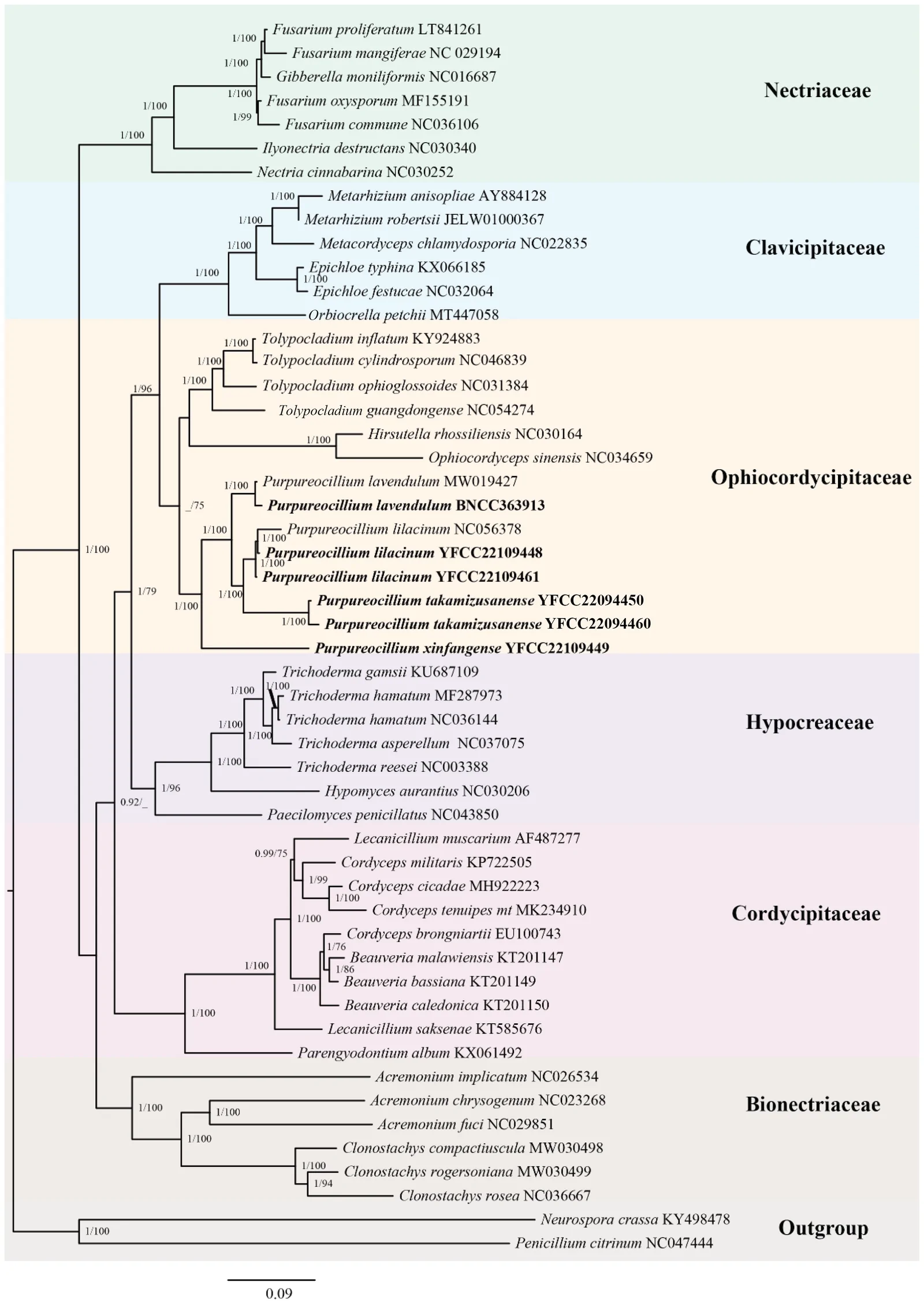
Phylogenetic tree reconstructed from concatenated amino acid sequences of 14 mitochondrial protein-coding genes (PCGs). Analyses were conducted under Bayesian inference (BI) and maximum likelihood (ML); node support values indicate Bayesian posterior probabilities (PP; left) and maximum likelihood bootstrap support values (BS; right), respectively. The scale bar represents 0.09 expected amino acid substitutions per site. *Neurospora crassa* and *Penicillium citrinum* were designated as outgroups. Taxa newly sequenced and emphasized in this study are highlighted in bold.

### Mitogenomic characteristics of the genus *Purpureocillium*

The mitochondrial genomes of four *Purpureocillium* species—*P. lilacinum*, *P. lavendulum*, *P. takamizusanense*, and *P. xinfangense*—displayed typical closed circular structures, as shown in **Figure 3** and summarized in **Table 3**. The respective genome sizes were 23,498 bp, 23,475 bp, 26,396 bp, and 121,341 bp. Thus, the mitochondrial genome of *P. xinfangense* was approximately four to five times larger than those of the other three species. Each of the four species contained 15 canonical protein-coding genes (PCGs), including ATP synthase subunit genes (*atp6*, *atp8*, *atp9*), the cytochrome b gene (*cob*), cytochrome c oxidase subunit genes (*cox1*, *cox2*, *cox3*), NADH dehydrogenase complex subunit genes (*nad1*, *nad2*, *nad3*, *nad4*, *nad4L*, *nad5*, *nad6*), and the ribosomal protein S3 gene (*rps3*). Additionally, all four species possessed 25 transfer RNA (tRNA) genes and two ribosomal RNA (rRNA) genes. Their GC contents were 28.5%, 28.6%, 27.2%, and 27.6%, respectively. AT skew was negative in *P. lilacinum*, *P. lavendulum*, and *P. takamizusanense* but positive in *P. xinfangense*, whereas GC skew values were positive in all four genomes (**Table 3**).

**Figure 3:**
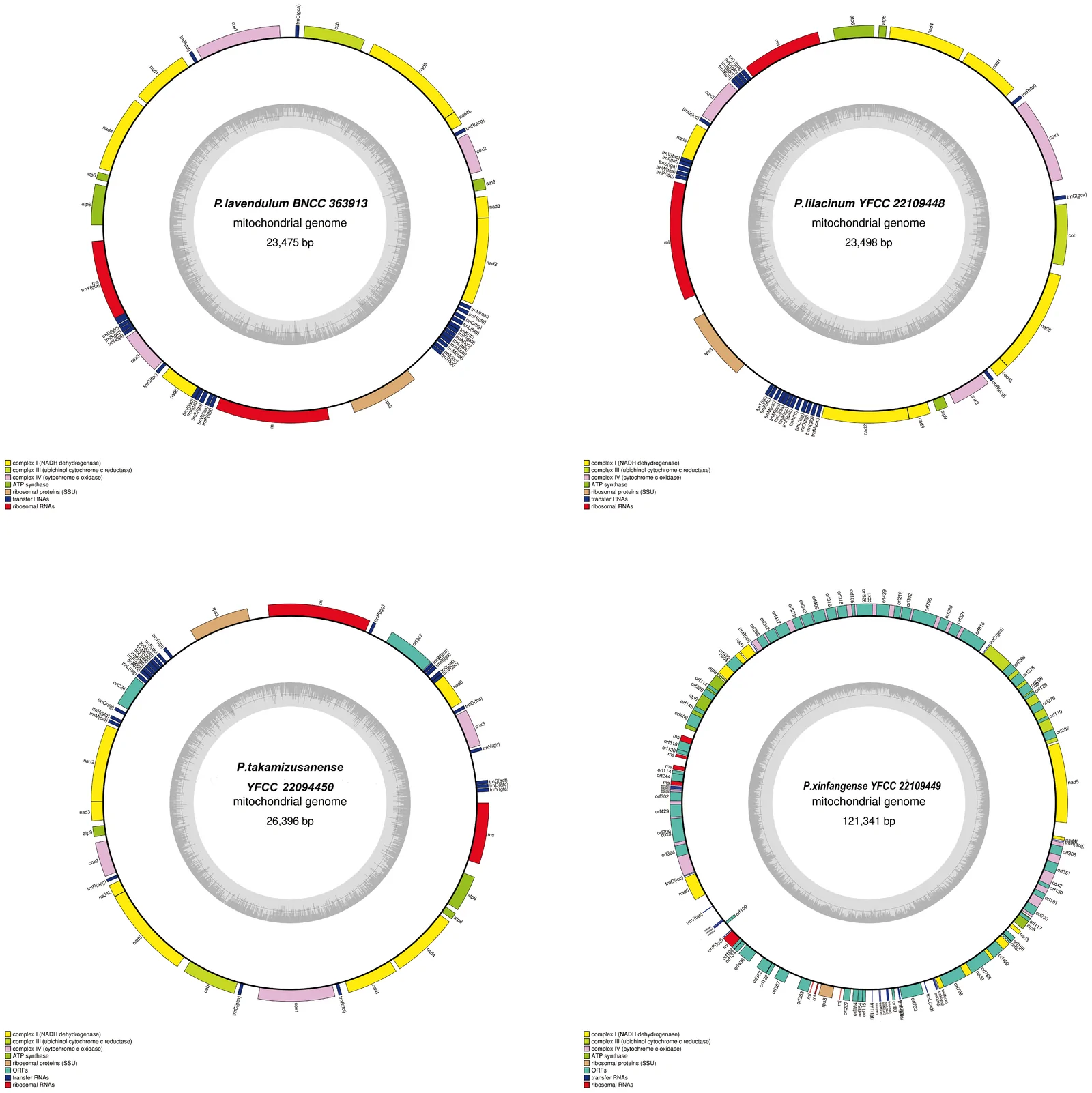
Circular maps of the complete mitochondrial genomes of *Purpureocillium lilacinum*, *P. lavendulum*, *P. takamizusanense*, and *P. xinfangense* generated with OGDRAW. Genes plotted outside and inside the main circle are transcribed in opposite directions, and colors correspond to functional gene categories indicated in the key. The inner grey rings represent GC-content graphs; radial ticks indicate genome positions.

**Table 3:** Mitochondrial genome characteristics of *Purpureocillium lilacinum*, *P. lavendulum*, *P. takamizusanense* and *P. xinfangense*.

Species	Genome size (bp)	PCGs (n)	GC content (%)	rRNA genes (n)	tRNA genes (n)	AT skew	GC skew	A+T (%)
*P. lilacinum* YFCC 22109448	23,498	15	28.4	2	25	-0.008	0.095	71.6
*P. lavendulum* BNCC 363913	23,475	15	28.5	2	25	-0.01	0.098	71.5
*P. takamizusanense* YFCC 22094450	26,396	15	27.2	2	25	-0.012	0.128	72.8
*P. xinfangense* YFCC 22109449	121,341	15	27.6	2	25	0.066	0.11	72.4

AT skew = (A − T)/(A + T); GC skew = (G − C)/(G + C). Positive and negative values indicate the direction of compositional asymmetry and are reported from the calculations used in this study.

### Morphological comparisons

The previously described species examined in this study were compared with the new taxon using expository text rather than formal taxonomic descriptions. *Purpureocillium lilacinum* was characterized by lilac colonies and subglobose to ellipsoidal conidia, *P. takamizusanense* by ellipsoidal conidia shorter than those of *P. lilacinum* but longer than those of *P. lavendulum*, and *P. lavendulum* by subglobose to lemon-shaped conidia with an apiculate base. In contrast, *P. xinfangense* produced long-cylindrical conidia and predominantly short-columnar, mucronate phialides, supporting its morphological distinction from the three previously described species.

### Phylogenetic analyses based on nuclear genes

Pairwise nucleotide comparisons between *Purpureocillium xinfangense* and closely related species revealed variable levels of sequence divergence across the five examined loci. The *ITS* regions showed 65 bp nucleotide differences between *P. xinfangense* and *P. lilacinum* and 69 bp differences between *P. xinfangense* and *P. lavendulum*, whereas comparisons with *P. takamizusanense* and *P. atypicola* could not be determined because of insufficient overlapping aligned regions. For the protein-coding loci, *P. xinfangense* differed from *P. lilacinum* by 44 bp in *TEF1-α* and 93 bp in *RPB1*, while differences with *P. lavendulum* reached 44–45 bp and 101–102 bp, respectively. The lowest divergence was observed between *P. xinfangense* and *P. atypicola*, with only 14 bp and 20 bp differences in *TEF1-α* and *RPB1*, respectively. Despite lower divergence in some conserved loci, the combined multilocus dataset consistently recovered *P. xinfangense* as a distinct lineage (see **Table 4**).

**Table 4:** Pairwise nucleotide differences were calculated based on available comparable aligned sites; comparisons lacking sufficient overlapping regions were indicated as “-”.

Species comparison	ITS	18S	28S	TEF1-α	RPB1
*P. xinfangense* vs *P. takamizusanense*	–	15 bp	20 bp	42 bp	100 bp
*P. xinfangense* vs *P. lilacinum*	65 bp	17 bp	24 bp	44 bp	93 bp
*P. xinfangense* vs *P. atypicola*	–	6 bp	1 bp	14 bp	20 bp
*P. xinfangense* vs *P. lavendulum*	69 bp	–	26 bp	44–45 bp	101–102 bp

### Morphological observations of previously described species

The three previously described species examined in this study showed morphological features generally consistent with earlier reports and were compared with the newly proposed species.

The examined culture of *Purpureocillium lilacinum* was obtained from Jinghong City, Puwen Township, Scales River, Yunnan Province, China, 100°58′51″E,22°51′34″N, 1000 m, 3 Oct 2022, Hong Yu, voucher YHHOPPW 2210065, living culture YFCC 22109448. After 14 d of incubation at 25 °C on MEA, colonies reached 58–61 mm in diameter. Colonies showed abundant white, densely tomentose mycelial growth. The central region was elevated, with sporulation initiating at the periphery, where the spore-producing area developed a mauve coloration. The reverse side gradually became lighter from the center outward, with a yellowish-brown center surrounded by a white peripheral ring. Mycelia were smooth and hyaline. Conidiophores measured 8.87–13.03 × 0.75–2.75 μm. Phialides were whorled and petaloid, with an expanded base tapering to a slender neck, 6.13–12.28 × 1.38–2.71 μm. Conidia were subglobose to ellipsoidal, forming chains, 1.46–2.46 × 1.05–2.43 μm. The colonies showed the characteristic lilac coloration of *P. lilacinum*, and chlamydospores were not observed.

The examined culture of *Purpureocillium takamizusanense* was collected from Yuanyang County, Nansha Township, Sharengou Village, Yunnan Province, China, 102°54′32″E, 23°18′18″N, 290 m, 24 Sep 2022, Hong Yu, voucher YHHOPYY 2209048, living culture YFCC 22094450. Colonies on MEA reached 51–53 mm in diameter after 14 d at 25 °C. Colonies initially appeared powdery and formed concentric circles after inoculation, subsequently developing a pinkish-purple hue radiating from the center after sporulation. Colony margins were white and well defined, and the reverse side was yellow, with the outermost ring remaining white. Mycelia were smooth, septate, and hyaline. Conidiophores measured 3.75–6.33 × 1.63–2.38 μm and bore umbrella-like phialides. Phialides were solitary or arranged in whorls of 2–6 along the conidiophore, 5.93–8.69 μm long, with an ellipsoid base 1.42–2.35 μm wide, gradually tapering into a distinct neck 0.34–0.79 μm diam. Conidia were ellipsoid, smooth-walled, hyaline, arranged in chains, and measured 1.91–2.54 × 1.12–1.98 μm. Compared with *P. lilacinum* and *P. lavendulum*, *P. takamizusanense* showed intermediate conidial dimensions.

The examined culture of *Purpureocillium lavendulum* was purchased from BeNa Biotech. After 7 d of incubation at 25 °C, colonies on MEA reached 30–32 mm in diameter and consisted of densely packed white hyphae. The reverse side of the colony was yellowish. Hyphae were hyaline and smooth-walled. Conidiophores measured 7.17–9.58 × 0.79–1.90 μm and showed irregular whorled branches, with terminal whorls consisting of 1–6 phialides. Phialides were elongated and cylindrical, tapering to a distinct neck, 6.88–11.61 × 1.08–1.59 μm, with the narrowest part 0.75–1.17 μm wide. Conidia were arranged in chains, subglobose to lemon-shaped, 1.68–2.63 × 1.08–1.83 μm, smooth-walled, and mostly translucent. The species was similar to *P. lilacinum* in its vinaceous colonies and conidial chains but differed by its slightly longer phialides and subglobose to limoniform catenate conidia with an apiculate base. It also produced a yellowish diffuse pigment and did not grow at 35 °C.

Overall, the three previously described species differed from *P. xinfangense* in conidial shape and phialide morphology. *Purpureocillium xinfangense* produced predominantly short-columnar, mucronate phialides and long-cylindrical conidia, whereas the examined and previously reported related species generally produced subglobose, ellipsoidal, lemon-shaped, or oval conidia.

## Taxonomy

*Purpureocillium lilacinum* (Thom) Luangsa-ard, Houbraken, Hywel-Jones & Samson.FEMS Microbiology Letters 321:141–149 (2011)

Basionym: *Penicillium lilacinum* Thom – Bull Bur Anim Ind US Dep Agric, 118: 73 (1910). = *Paecilomyces lilacinus* (Thom) Samson – Stud Mycol 6: 58 (1974).

= *Paecilomyces nostocoides* Dunn – Mycologia 75: 179 (1983).

**Figure 4**.

**Figure 4:**
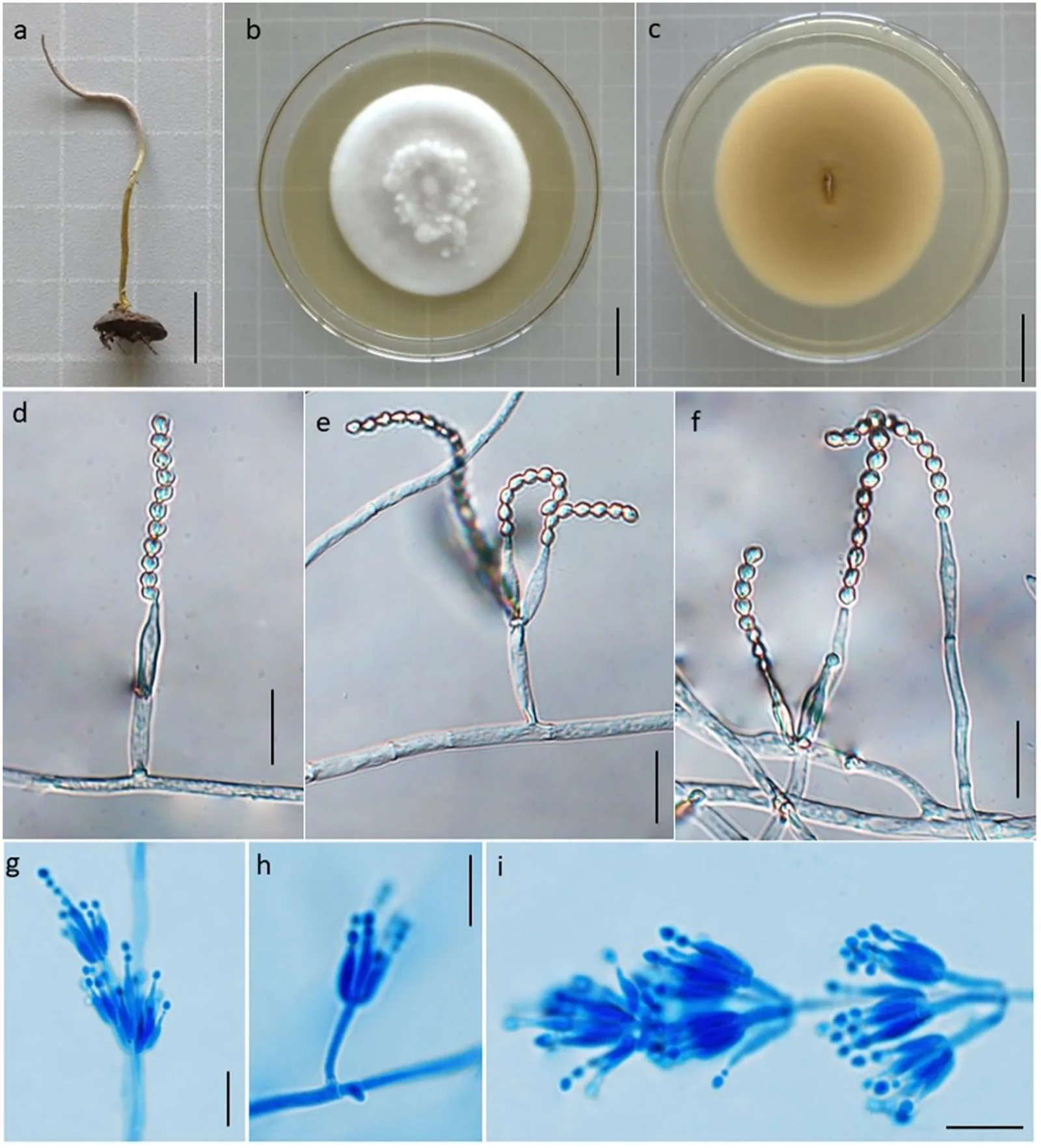
Morphological characteristics of *Purpureocillium lilacinum* (YFCC 22109448). **(a)** Fungal association with Hemiptera; **(b, c)**. Colony morphology on MEA medium after incubation at 25 °C for 14 days; **(d–i)**. Phialides and conidia morphology. Scale bars: **(a–c)** = 20 mm; d–i = 10 μm.

Description:After 14 days of incubation at 25 °C on Malt Extract Agar (MEA), the colony attained a diameter of 58.0–61.0 mm. The colony was characterized by abundant, white, and densely tomentose mycelium, with a raised central region. Sporulation initiated at the colony periphery, where the spore-producing area developed a mauve pigmentation. The reverse of the colony exhibited a color gradient, lightening from the center outward, with a yellowish-brown central zone surrounded by a white peripheral ring. The hyphae were smooth and hyaline. Conidiophores measured 8.87–13.03 × 0.75–2.75 μm. Phialides were arranged in whorls, appearing petaloid, with an expanded base and a tapering apical neck, measuring 6.13–12.28 × 1.38–2.71 μm. Conidia were subglobose to ellipsoidal, formed in chains, and measured 1.46–2.46 × 1.05–2.43 μm.

Distribution: worldwide.

Examined specimens: China, Yunnan Province, Jinghong City, Puwen Township, Chenggan River (100°58′51″E, 22°51′34″N; elevation: 1,000 m a.s.l.). Specimen was collected from Hemiptera larvae in a Monsoon evergreen broadleaf forest, on 3 October 2022 by Hong Yu. Holotype specimen deposited as YHHOPPW 2210065; ex-type living culture preserved as YFCC 22109448.

Notes: Colonies of *Purpureocillium lilacinum* exhibit a characteristic lilac pigmentation; the aerial mycelium is hyaline and verticillately arranged, with each whorl bearing 1–6 phialides. Growth occurs over a temperature range of 8–38 °C, with an optimal range of 26–30 °C. This soil-inhabiting fungus displays a broad host spectrum, primarily parasitizing nematodes and other soil-dwelling invertebrates. The specimen was isolated from larvae of Hemiptera collected in a monsoon evergreen broadleaf forest.

*Purpureocillium takamizusanense* S. Ban, Azuma & Hirok. Sato.

International Journal of Systematic and Evolutionary Microbiology 65: 2459–2465 (2015)

Basionym: *Isaria takamizusanensis* Kobayasi, Sci. Rep. Tokyo Bunrika Daig., Sect. B 5: 229, 1941 Synonym: *Cordyceps ryogamimontana* Kobayasi & Shimizu, Bull. Nat. Sci. Mus., Tokyo 6: 303, 1963.

**Figure 5**.

**Figure 5:**
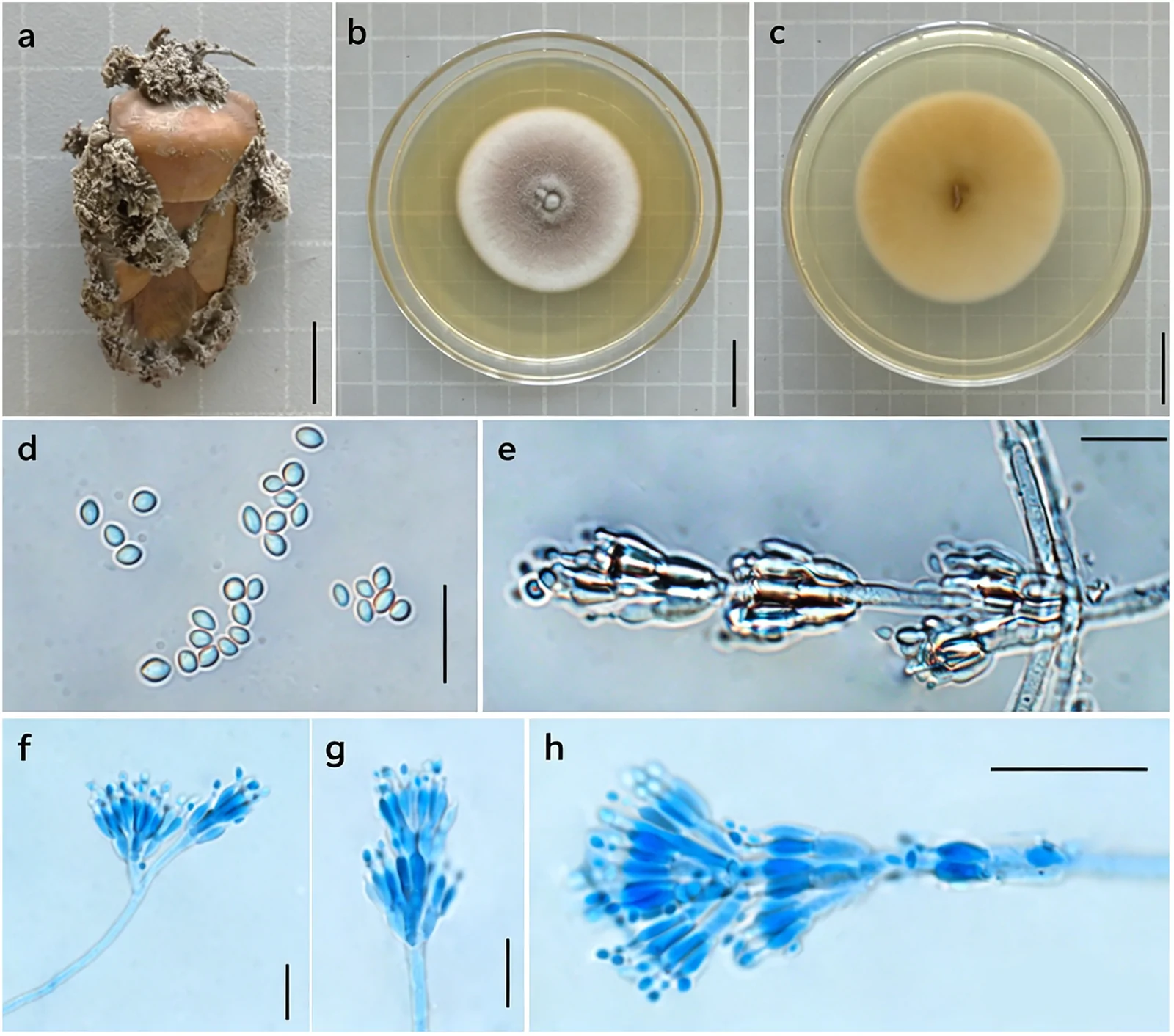
Morphological characteristics of Purpureocillium takamizusanense (YFCC 22094450). **(a)** Fungal growth on Hemiptera; **(b)** Colony morphology on PDA medium after incubation at 25 °C for 7 days; **(c–h)**. Phialides and conidia. Scale bars: a–c = 20 mm; d–h = 10 mm.

Description:Colonies on malt extract agar (MEA) attained diameters of 51–53 mm after 14 days of incubation at 25 °C. Colony texture was powdery, exhibiting concentric zonation upon initial growth; pigmentation developed progressively from the centre outward following conidiation, resulting in a pinkish-purple hue. Margins were white and regular; the reverse side displayed uniform yellow pigmentation. The outermost zone remained white. Hyphae were smooth-walled, septate, and hyaline. Conidiophores measured 3.75–6.33 × 1.63–2.38 μm, bearing umbrella-like phialides either singly or in whorls of 2–6. Phialides were ellipsoid at the base (5.93–8.69 μm long, 1.42–2.35 μm wide), tapering gradually into a distinct neck (0.34–0.79 μm in diameter). Conidia were produced in unbranched chains, ellipsoidal, smooth-walled, hyaline, and measured 1.91–2.54 × 1.12–1.98 μm.

Distribution: Japan, China

Specimens examined: China, Yunnan Province, Yuanyang County, Nansha Township, Sharengou Village (102°54′32″ E,23°18′18″ N; elevation: 290 m a.s.l.). Specimen collected from Hemiptera larvae in soil of a lychee orchard within the Red River Hot and Dry Valley, on 24 September 2022 by Hong Yu. Holotype voucher deposited under accession number YHHOPYY 2209048; ex-type living culture preserved as YFCC 22094450.

Notes: Axenic cultures of *Purpureocillium lilacinum* and *P. lavendulum* are typically isolated from soil, whereas *P. takamizusanense* is predominantly recovered from insect hosts in the families Cicadidae and Pentatomidae. The isolates examined in this study were obtained from field-collected Hemiptera larvae sampled in a lychee orchard located in the Red River Hot and Dry Valley. Microscopic examination revealed morphological features consistent with those of *P. lilacinum* and *P. lavendulum*, including globose to subglobose phialides producing unbranched chains of ellipsoidal, hyaline conidia.

*Purpureocillium lavendulum* H. Perdomo, Gené, Cano & Guarro. Mycologia 105: 151–161 (2013)

**Figure 6**.

**Figure 6:**
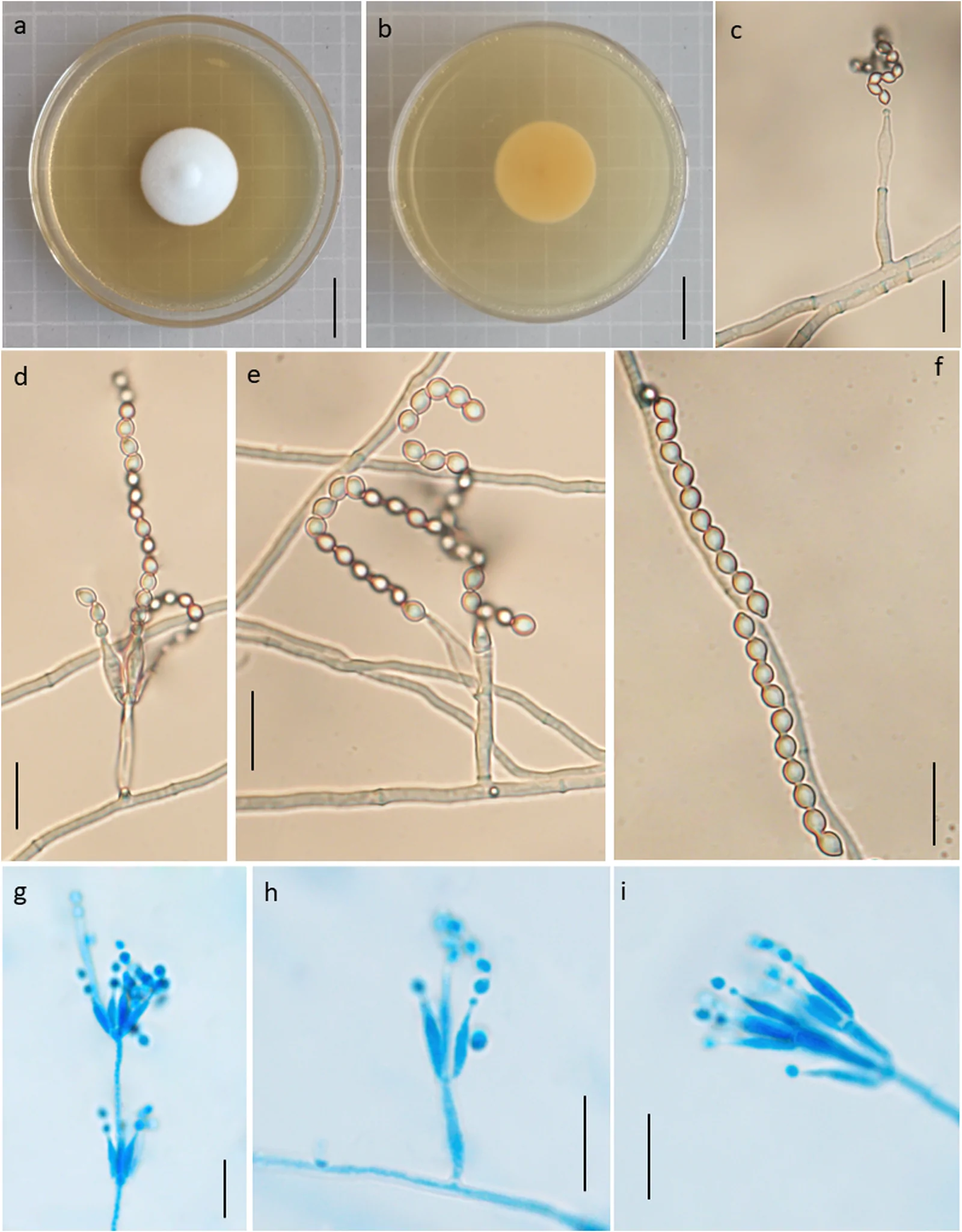
Morphological characteristics of *Purpureocillium lavendulum* (BNCC 363913). **(a, b)**. Colony morphology on PDA medium after incubation at 25 °C for 7 days; **(c–i)**. Morphology of phialides and conidia. Scale bars: **(a, b)** = 20 mm; c–i = 10 mm.

Description: After 7 days of incubation at 25 °C on malt extract agar (MEA), colonies attained a diameter of 30–32 mm and consisted of dense, white aerial hyphae; the reverse side appeared pale yellow. Hyphae were hyaline with smooth walls. Conidiophores measured 7.17–9.58 × 0.79–1.90 μm, bearing irregularly verticillate branches; terminal whorls comprised 1–6 phialides, each cylindrical and gradually tapering into a distinct neck measuring 6.88–11.61 × 1.08–1.59 μm, with a basal width of 0.75–1.17 μm. Conidia were produced in unbranched chains, subglobose to lemon-shaped, measuring 1.68–2.63 × 1.08–1.59 μm (length × width), with smooth, translucent walls.

Distribution: Worldwide.

Specimens examined: BNCC 363913 (= CBS 128677 = FMR 10376)

Notes: *Purpureocillium lavendulum* is phylogenetically closely related to *P. lilacinum*, yet exhibits distinct morphological and physiological traits. These include denser aerial mycelium; subglobose to lemon-shaped conidia bearing apiculate necks—slightly longer than those of *P. lilacinum*; secretion of a diffusible yellow pigment; and failure to grow at 35 °C.

*Purpureocillium xinfangense* Hong Yu bis & L.J. Luo, sp. nov. **Figure 7**.

**Figure 7:**
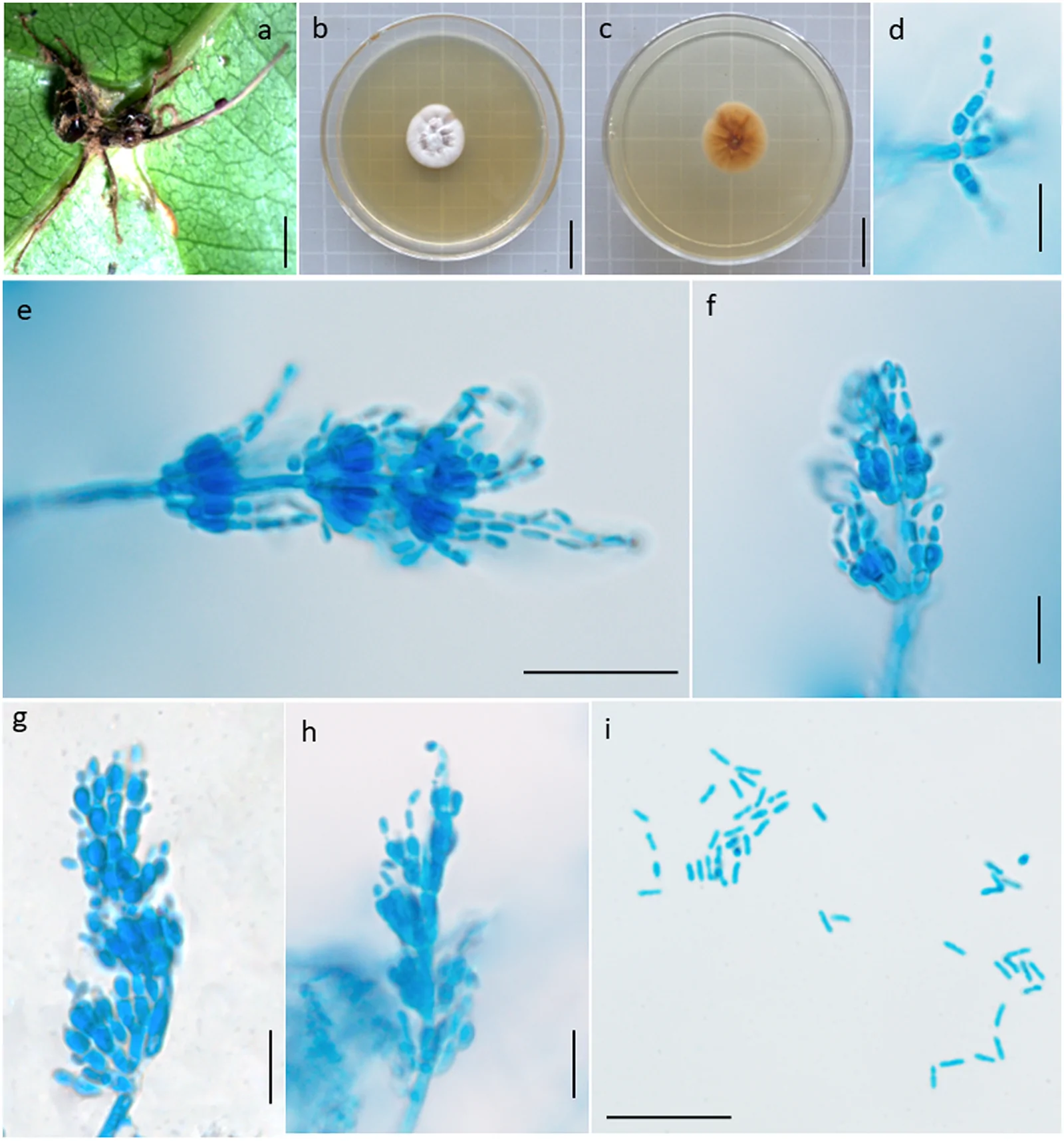
Morphology of *Purpureocillium xinfangense* (YFCC 22109449). **(a)** Fungus associated with an unidentified ant cadaver; **(b, c)**. Colony characteristics on PDA medium after incubation at 25 °C for 14 days; **(d–h)**. Phialides and conidia morphology; **(i)**. Conidia details. Scale bars: **(a–c)** = 10 mm; **(e, i)** = 20 μm; **(d, f–h)** = 10 μm.

MycoBank: MB856649

Typification: China, Yunnan Province, Pu’er City, Nanping County, Xinfang Reservoir (100°57′43″E,22°42′19″N; elevation: 1380 m a.s.l.). Specimen collected from a naturally infected, unidentified ant cadaver associated with an unidentified *Ophiocordyceps unilateralis* species complex, 24 September 2022, by Hong Yu. Neither morphological nor molecular identification was performed for the ant host or the associated *Ophiocordyceps unilateralis* species complex. Holotype deposited as accession number YHH 2210040; ex-type living culture preserved as accession number YFCC 22109449.

Etymology: The specific epithet “xinfangense” is derived from the Xinfang Reservoir, the type locality of this species, located in Nanping County, Pu’er City, Yunnan Province, China.

Diagnosis: The conidia of *P. xinfangense* exhibit a cylindrical morphology, whereas the conidia of other species within this genus typically display a lemon-shaped or elliptical configuration.

Description: Asexual morph. After 14 d of incubation on MEA at 25 °C, the colony diameter ranged from 24 to 26 mm. The growth rate was moderate, with the colony surface exhibiting white fluffy mycelium and a pink-purple sporulation zone. The reverse coloration gradually lightened from the center outward, transitioning to an orange-yellow wrinkled appearance. The hyphae were hyaline, branched, and smooth-walled. Conidiophores were solitary, measuring 3.17–6.26 × 1.96–3.15 μm. Phialides were whorled, with 2–3 per conidiophore, predominantly short-columnar and mucronate, measuring 3.17–5.26 × 2.16–2.70 μm. Conidia were long-cylindrical, forming chains, and measured 2.62–4.67 × 0.65–1.59 μm.

Distribution: China, Yunnan Province, Pu’er City, Nanping County, Xinfang Reservoir.

Notes: *Purpureocillium xinfangense* was isolated from an ant cadaver whose host species and associated *Ophiocordyceps unilateralis* species complex remain unidentified. Consequently, the ecological association is reported conservatively, with no species-level taxonomic assignments made for either the host ant or the fungal pathogen. The type strain of *P. lilacinum* was predominantly isolated from soil (Luangsa-Ard et al., 2011). Previous studies have reported that the insect hosts of *P. takamizusanense* include members of Cicadidae and Pentatomidae (Hemiptera) (Ban et al., 2015). *Purpureocillium atypicola* is associated with spiders (Yasuda, 1915, 1917; Spatafora et al., 2015). Moreover, the conidia of *P. xinfangense* displayed a distinctly elongated cylindrical morphology, contrasting with the lemon-shaped or oval conidia typical of several previously described species (Luangsa-Ard et al., 2011; Perdomo et al., 2013; Ban et al., 2015). Other specimens examined: No additional specimens were examined.

Among the 15 canonical protein‐coding genes in the mitochondrial genomes of four *Purpureocillium* species, *nad5* was the longest (2007 bp) and *atp8* the shortest (147 bp). Each species contained 25 tRNA genes ranging from 71 to 87 bp in length. The lengths of rRNA genes varied among species: *rnl* ranged from 1316 to 2265 bp (2265 bp in *P. lilacinum*, 1518 bp in *P. lavendulum*, 1316 bp in *P. takamizusanense*, and 1588 bp in *P. xinfangense*), while *rns* ranged from 1518 to 1772 bp (1519 bp in *P. lilacinum*, 1518 bp in *P. lavendulum*, and 1772 bp in both *P. takamizusanense* and *P. xinfangense*). The protein‐coding genes of the four species mainly used TTG and ATG as start codons, with TAG and TAA as the predominant stop codons. Detailed annotations including gene coordinates, feature lengths, intergenic spacers, and gene overlaps of the mitochondrial genomes are provided in **Supplementary Table S1**, in which intergenic spacer lengths are presented as non‐negative values and gene overlaps are listed separately instead of being represented by negative spacer values. The main mitochondrial genome characteristics of the four *Purpureocillium* species are summarized in **Table 5**.

**Table 5:** Summary of mitochondrial genome characteristics of four *Purpureocillium* species.

Species	Genome size (bp)	GC content (%)	Protein-coding genes	tRNA genes	rRNA genes
*P. lilacinum* YFCC 22109448	23,498	28.5	15	25	2
*P. lavendulum* BNCC 363913	23,475	28.6	15	25	2
*P. takamizusanense* YFCC 22094450	26,396	27.2	15	25	2
*P. xinfangense* YFCC 22109449	121,341	27.6	15	25	2

## Discussion

*Purpureocillium xinfangense* is supported as a distinct taxon based on the combined evidence of morphology and multilocus phylogenetic analyses. It formed an independent and strongly supported lineage in both the concatenated nuclear-gene and mitochondrial datasets. Morphologically, *P. xinfangense* differs from the compared congeners by its elongated cylindrical conidia and predominantly short-columnar, mucronate phialides. This integrative evidence is particularly important because recent taxonomic studies have considerably expanded the known morphological, ecological, and phylogenetic diversity of *Purpureocillium* (Araújo et al., 2025; Chen et al., 2024b). Similar to these recently described species, the recognition of *P. xinfangense* is based on the integration of morphological differentiation and multilocus phylogenetic evidence. The clustering of *P. sodanum* and *P. zongqii* with *P. lilacinum* may reflect their close evolutionary affinity, although their precise phylogenetic positions require further evaluation due to the limited availability of sequence data and the absence of corresponding voucher specimens.

The ecological association of *P. xinfangense* also provides an interesting perspective. Hyperparasitism represents an important fungal interaction in which one fungus colonizes or obtains resources from another fungus, potentially influencing fungal community structure and host–pathogen dynamics. In this study, *P. xinfangense* was isolated from an ant-associated fungal substrate, suggesting a possible ecological association with entomopathogenic fungi (Araújo et al., 2025; Luangsa-Ard et al., 2011). However, because the associated fungus was not formally identified to species level, the exact ecological relationship remains uncertain. Further studies involving direct observation, infection assays, and genomic analyses are required to determine whether *P. xinfangense* exhibits a true hyperparasitic lifestyle.

The nuclear and mitochondrial phylogenetic analyses showed largely congruent patterns and consistently recovered *P. xinfangense* as an independent lineage within *Purpureocillium*. The mitochondrial protein-coding genes provided additional phylogenetic information and supported the evolutionary placement of the new species. Nevertheless, recent species discoveries indicate that species delimitation within *Purpureocillium* should rely on multiple independent lines of evidence (Araújo et al., 2025; Chen et al., 2024b; Taylor et al., 2000). Expanded taxon sampling and additional genomic resources will be important for further evaluating species boundaries and evolutionary relationships within this genus.

The most conspicuous mitochondrial feature of *P. xinfangense* is its 121,341 bp mitogenome, which differs drastically from the approximately 23–26 kb mitogenomes of the other three species examined in this study. At present, the relative contributions of intron proliferation, repeat accumulation, and the acquisition or retention of additional open reading frames cannot be resolved solely on the basis of genome size. Chang et al. (2024) reported a 112,465 bp mitogenome for *P. atypicola* that contains numerous introns, and demonstrated that intron dynamics account for the majority of size variation among the four *Purpureocillium* mitogenomes included in their comparative analysis. Analogous mechanisms are widely observed across fungal taxa (Burger et al., 2003; Aguileta et al., 2014): intron invasion drove substantial mitochondrial genome expansion in *Rhynchosporium* (Torriani et al., 2014), and the 135 kb mitogenome of *Agaricus bisporus* (J.E. Lange) Imbach harbors extensive group I/II introns and plasmid-related sequences (Férandon et al., 2013). A kingdom-wide survey similarly found that length variation in fungal mitogenomes is strongly associated with accessory genetic elements such as introns, homing endonuclease genes, and unidentified ORFs (Fonseca et al., 2021). These comparisons generate testable explanations for the expanded mitogenome of *P. xinfangense*, yet repeat annotation, intron mapping, and classification of accessory ORFs are required before a dominant mechanism can be definitively confirmed.

Nucleotide-composition asymmetry further provides insights into mitochondrial evolution. In the present study, *P. lilacinum*, *P. lavendulum*, and *P. takamizusanense* showed slightly negative AT skew values, whereas *P. xinfangense* exhibited a positive AT skew value. In contrast, GC skew values were positive in all four examined genomes. Therefore, the major compositional difference distinguishing *P. xinfangense* involves AT skew rather than GC skew. Such strand-specific compositional biases may be associated with asymmetric mutational pressures during mitochondrial replication and transcription (Rocha, 2004; Sueoka, 1995); however, additional comparative mitogenomic data are required to clarify their evolutionary significance.

Recent taxonomic advances further emphasize the necessity of extensive comparative sampling when proposing new *Purpureocillium* species. Since 2024, five new species of the genus *Purpureocillium* have been formally described: *P. jiangxiense* and *P. zongqii* (Chen et al., 2024a, b), based on integrated morphological and multilocus phylogenetic analyses; *P. araneae*, *P. sinense*, and *P. tiankengense* (Chen et al., 2025), delineated as novel taxa from China; *P. atlanticum* (Araújo et al., 2025), characterized through an integrative taxogenomic framework informed by its association with trapdoor spiders in Brazil; and *P. araneicola* in China (Chang et al., 2026), validated using morphological data and a five-locus phylogenetic reconstruction. These recently described taxa expand the documented phenotypic and ecological range of the genus, and provide critical contextual support for the assessment of *P. xinfangense*. Future analyses should incorporate these lineages directly into expanded multilocus and genomic phylogenies once adequate sequence coverage becomes available.

Since the establishment of the genus *Purpureocillium* (*Ophiocordycipitaceae*) in 2011 (Luangsa-Ard et al., 2011), only a limited number of species have been formally described. *Purpureocillium* is a globally distributed, morphologically and ecologically diverse anamorphic fungal genus with significant economic relevance in industrial, pharmaceutical, and environmental applications. To date, research on this genus has been largely focused on two well-characterized species—*P. lilacinum* and *P. lavendulum* (Luangsa-Ard et al., 2011; Perdomo et al., 2013)—while taxonomic, genomic, and functional studies of other described members remain scarce. Consequently, future efforts should prioritize broadened taxonomic sampling across the genus to enable comprehensive species delimitation and systematic evaluation of the biocontrol potential of its constituent lineages.

## Data Availability

The original contributions presented in the study are publicly available. This data can be found here: NCBI GenBank, accession PRJNA1539032, SAMN63984444.
