# Supplementary material for: Phylogenetic reconstruction of the genus Purpureocillium based on multilocus nuclear and mitochondrial DNA sequences, with the description of Purpureocillium xinfangense sp. nov. (Ophiocordycipitaceae, Hypocreales) from Southwestern China
Source: Front Fungal Biol. 2026 Oct 13;7:1972355. doi: 10.3389/ffunb.2026.1972355 (PMC13710243; doi:10.3389/ffunb.2026.1972355)
Supplement: Supplementary file 1 [file Table1.docx]

**Supplementary Table S1.**

Detailed mitochondrial genome annotation of four *Purpureocillium* species

| **Species** | **Gene** | | **Region** | | | | **Length (bp)** | | **Adjacent gene distance (bp)** | | **Start codon** | | **Stop codon** | |  |
| --- | --- | --- | --- | --- | --- | --- | --- | --- | --- | --- | --- | --- | --- | --- | --- |
|  |  |  | **From** | | **To** | |  |  |  |  |  |  |  |  |  |
| *Purpureocillium lilacinum* | | *trnC(gca)* | | 478 | | 549 | | 72 | | 477 | |  | |  | |
|  |  | *cox1* | | 848 | | 2,488 | | 1,641 | | 298 | | TTG | | TAG | |
|  |  | *trnR(tct)* | | 2,576 | | 2,646 | | 71 | | 87 | |  | |  | |
|  |  | *nad1* | | 2,808 | | 3,917 | | 1,110 | | 161 | | ATG | | TAA | |
|  |  | *nad4* | | 4,007 | | 5,464 | | 1,458 | | 89 | | ATG | | TAG | |
|  |  | *atp8* | | 5,536 | | 5,682 | | 147 | | 71 | | ATG | | TAA | |
|  |  | *atp6* | | 5,771 | | 6,550 | | 780 | | 88 | | ATG | | TAA | |
|  |  | *rns* | | 6,846 | | 8,364 | | 1,519 | | 295 | |  | |  | |
|  |  | *trnY(gta)* | | 8,407 | | 8,490 | | 84 | | 42 | |  | |  | |
|  |  | *trnD* | | 8,502 | | 8,574 | | 73 | | 11 | |  | |  | |
|  |  | *trnS(gct)* | | 8,580 | | 8,661 | | 82 | | 5 | |  | |  | |
|  |  | *trnN(gtt)* | | 8,666 | | 8,736 | | 71 | | 4 | |  | |  | |
|  |  | *cox3* | | 8,779 | | 9,588 | | 810 | | 42 | | ATG | | TAA | |
|  |  | *trnG(tcc)* | | 9,624 | | 9,694 | | 71 | | 35 | |  | |  | |
|  |  | *nad6* | | 9,777 | | 10,499 | | 723 | | 82 | | ATG | | TAA | |
|  |  | *trnV(tac)* | | 10,450 | | 10,522 | | 73 | | -50 | |  | |  | |
|  |  | *trnI(gat)* | | 10,524 | | 10,595 | | 72 | | 1 | |  | |  | |
|  |  | *trnS(tga)* | | 10,617 | | 10,703 | | 87 | | 21 | |  | |  | |
|  |  | *trnW(tca)* | | 10,720 | | 10,791 | | 72 | | 16 | |  | |  | |
|  |  | *trnP(tgg)* | | 10,800 | | 10,872 | | 73 | | 8 | |  | |  | |
|  |  | *rnl* | | 10,941 | | 13,205 | | 2,265 | | 68 | |  | |  | |
|  |  | *rps3* | | 13,602 | | 14,912 | | 1,311 | | 396 | | ATG | | TAA | |
|  |  | *trnT(tgt)* | | 15,570 | | 15,640 | | 71 | | 657 | |  | |  | |
|  |  | *trnE* | | 15,648 | | 15,719 | | 72 | | 7 | |  | |  | |
|  |  | *trnM(cat)* | | 15,755 | | 15,826 | | 72 | | 35 | |  | |  | |
|  |  | *trnM(cat)_copy2* | | 15,850 | | 15,922 | | 73 | | 23 | |  | |  | |
|  |  | *trnL(taa)* | | 15,925 | | 16,006 | | 82 | | 2 | |  | |  | |
|  |  | *trnA(tgc)* | | 16,012 | | 16,084 | | 73 | | 5 | |  | |  | |
|  | | *trnF(gaa)* | | 16,091 | | 16,163 | | 73 | | 6 | |  | |  | |
|  |  | *trnK(ttt)* | | 16,199 | | 16,271 | | 73 | | 35 | |  | |  | |
|  |  | *trnL(tag)* | | 16,316 | | 16,397 | | 82 | | 44 | |  | |  | |
|  |  | *trnQ(ttg)* | | 16,412 | | 16,484 | | 73 | | 14 | |  | |  | |
|  |  | *trnH(gtg)* | | 16,514 | | 16,587 | | 74 | | 29 | |  | |  | |
|  |  | *trnM(cat)_copy3* | | 16,619 | | 16,690 | | 72 | | 31 | |  | |  | |
|  |  | *nad2* | | 16,728 | | 18,392 | | 1,665 | | 37 | | ATG | | TAA | |
|  |  | *nad3* | | 18,393 | | 18,806 | | 414 | |  | | ATG | | TAA | |
|  |  | *atp9* | | 18,928 | | 19,152 | | 225 | | 121 | | ATG | | TAA | |
|  |  | *cox2* | | 19,290 | | 20,039 | | 750 | | 137 | | ATG | | TAA | |
|  |  | *trnR(acg)* | | 20,095 | | 20,165 | | 71 | | 55 | |  | |  | |
|  |  | *nad4L* | | 20,250 | | 20,519 | | 270 | | 84 | | ATG | | TAA | |
|  |  | *nad5* | | 20,519 | | 22,525 | | 2,007 | | -1 | | ATG | | TAA | |
|  |  | *cob* | | 22,713 | | 23,498 | | 1,170 | |  | | ATG | | TAA | |
| *Purpureocillium lavendulum* | | *nad2* | | 21,931 | | 23,475 | | 1,665 | |  | | ATG | | TAA | |
|  |  |  | | 1 | | 120 | |  | |  | |  | |  | |
|  |  | *nad3* | | 121 | | 534 | | 414 | | 120 | | ATG | | TAA | |
|  |  | *atp9* | | 656 | | 880 | | 225 | | 121 | | ATG | | TAA | |
|  |  | *cox2* | | 1,015 | | 1,764 | | 750 | | 134 | | ATG | | TAA | |
|  |  | *trnR(acg)* | | 1,819 | | 1,889 | | 71 | | 54 | |  | |  | |
|  |  | *nad4L* | | 1,974 | | 2,243 | | 270 | | 84 | | ATG | | TAA | |
|  |  | *nad5* | | 2,243 | | 4,249 | | 2,007 | | -1 | | ATG | | TAA | |
|  |  | *cob* | | 4,429 | | 5,598 | | 1,170 | | 179 | | ATG | | TAA | |
|  |  | *trnC(gca)* | | 5,697 | | 5,768 | | 72 | | 98 | |  | |  | |
|  |  | *cox1* | | 6,064 | | 7,704 | | 1,641 | | 295 | | TTG | | TAG | |
|  |  | *trnR(tct)* | | 7,791 | | 7,861 | | 71 | | 86 | |  | |  | |
|  |  | *nad1* | | 8,011 | | 9,120 | | 1,110 | | 149 | | ATG | | TAA | |
|  |  | *nad4* | | 9,210 | | 10,667 | | 1,458 | | 89 | | ATG | | TAA | |
|  | | *atp8* | | 10,742 | | 10,888 | | 147 | | 74 | | ATG | | TAA | |
|  |  | *atp6* | | 10,975 | | 11,754 | | 780 | | 86 | | ATG | | TAA | |
|  |  | *trnY(gta)* | | 12,057 | | 13,706 | | 1,650 | | 302 | |  | |  | |
|  |  | *rns* | | 12,063 | | 13,580 | | 1,518 | | -1,644 | |  | |  | |
|  |  | *trnD* | | 13,718 | | 13,790 | | 73 | | 137 | |  | |  | |
|  |  | *trnS(gct)* | | 13,796 | | 13,877 | | 82 | | 5 | |  | |  | |
|  |  | *trnN(gtt)* | | 13,882 | | 13,952 | | 71 | | 4 | |  | |  | |
|  |  | *cox3* | | 13,995 | | 14,804 | | 810 | | 42 | | ATG | | TAA | |
|  |  | *trnG(tcc)* | | 14,840 | | 14,910 | | 71 | | 35 | |  | |  | |
|  |  | *nad6* | | 14,993 | | 15,715 | | 723 | | 82 | | ATG | | TAA | |
|  |  | *trnV(tac)* | | 15,666 | | 15,738 | | 73 | | -50 | |  | |  | |
|  |  | *trnI(gat)* | | 15,740 | | 15,811 | | 72 | | 1 | |  | |  | |
|  |  | *trnS(tga)* | | 15,833 | | 15,919 | | 87 | | 21 | |  | |  | |
|  |  | *trnW(tca)* | | 15,962 | | 16,033 | | 72 | | 42 | |  | |  | |
|  |  | *trnP(tgg)* | | 16,042 | | 16,114 | | 73 | | 8 | |  | |  | |
|  |  | *rnl* | | 16,180 | | 18,349 | | 2,170 | | 65 | |  | |  | |
|  |  | *rps3* | | 18,833 | | 20,140 | | 1,308 | | 483 | | ATG | | TAA | |
|  |  | *trnT(tgt)* | | 20,796 | | 20,866 | | 71 | | 655 | |  | |  | |
|  |  | *trnE* | | 20,873 | | 20,944 | | 72 | | 6 | |  | |  | |
|  |  | *trnM(cat)* | | 20,973 | | 21,043 | | 71 | | 28 | |  | |  | |
|  |  | *trnM(cat)_copy2* | | 21,061 | | 21,133 | | 73 | | 17 | |  | |  | |
|  |  | *trnL(taa)* | | 21,136 | | 21,217 | | 82 | | 2 | |  | |  | |
|  |  | *trnA(tgc)* | | 21,223 | | 21,295 | | 73 | | 5 | |  | |  | |
|  | | *trnF(gaa)* | | 21,304 | | 21,376 | | 73 | | 8 | |  | |  | |
|  |  | *trnK(ttt)* | | 21,377 | | 21,449 | | 73 | |  | |  | |  | |
|  |  | *trnL(tag)* | | 21,482 | | 21,563 | | 82 | | 32 | |  | |  | |
|  |  | *trnQ(ttg)* | | 21,588 | | 21,660 | | 73 | | 24 | |  | |  | |
|  |  | *trnH(gtg)* | | 21,705 | | 21,778 | | 74 | | 44 | |  | |  | |
|  |  | *trnM(cat)_copy3* | | 21,812 | | 21,883 | | 72 | | 33 | |  | |  | |
| *Purpureocillium takamizusanense* | | *trnY(gta)* | | 235 | | 318 | | 84 | | 234 | |  | |  | |
|  |  | *trnD* | | 327 | | 399 | | 73 | | 8 | |  | |  | |
|  |  | *trnS(gct)* | | 405 | | 486 | | 82 | | 5 | |  | |  | |
|  |  | *trnN(gtt)* | | 1,118 | | 1,189 | | 72 | | 631 | |  | |  | |
|  |  | *cox3* | | 1,232 | | 2,041 | | 810 | | 42 | | ATG | | TAA | |
|  |  | *trnG(tcc)* | | 2,077 | | 2,147 | | 71 | | 35 | |  | |  | |
|  |  | *nad6* | | 2,229 | | 2,948 | | 720 | | 81 | | ATG | | TAA | |
|  |  | *trnV(tac)* | | 2,899 | | 2,971 | | 73 | | -50 | |  | |  | |
|  |  | *trnI(gat)* | | 2,973 | | 3,044 | | 72 | | 1 | |  | |  | |
|  |  | *trnS(tga)* | | 3,121 | | 3,207 | | 87 | | 76 | |  | |  | |
|  |  | *trnW(tca)* | | 3,216 | | 3,287 | | 72 | | 8 | |  | |  | |
|  |  | *trnP(tgg)* | | 4,719 | | 4,790 | | 72 | | 379 | |  | |  | |
|  |  | *rnl* | | 4,862 | | 7,076 | | 2,215 | | 71 | |  | |  | |
|  |  | *rps3* | | 7,509 | | 8,798 | | 1,290 | | 432 | | ATG | | TAA | |
|  |  | *trnT(tgt)* | | 9,433 | | 9,503 | | 71 | | 634 | |  | |  | |
|  |  | *trnE* | | 9,614 | | 9,685 | | 72 | | 110 | |  | |  | |
|  |  | *trnM(cat)* | | 9,717 | | 9,788 | | 72 | | 31 | |  | |  | |
|  |  | *trnM(cat)_copy2* | | 9,802 | | 9,874 | | 73 | | 13 | |  | |  | |
|  |  | *trnL(taa)* | | 9,877 | | 9,958 | | 82 | | 2 | |  | |  | |
|  |  | *trnA(tgc)* | | 9,964 | | 10,036 | | 73 | | 5 | |  | |  | |
|  |  | *trnF(gaa)* | | 10,044 | | 10,116 | | 73 | | 7 | |  | |  | |
|  |  | *trnK(ttt)* | | 10,117 | | 10,189 | | 73 | |  | |  | |  | |
|  |  | *trnL(tag)* | | 10,222 | | 10,303 | | 82 | | 32 | |  | |  | |
|  |  | *trnQ(ttg)* | | 11,083 | | 11,155 | | 73 | | 79 | |  | |  | |
|  |  | *trnH(gtg)* | | 11,281 | | 11,354 | | 74 | | 125 | |  | |  | |
|  | | *trnM(cat)_copy3* | | 11,381 | | 11,452 | | 72 | | 26 | |  | |  | |
|  |  | *nad2* | | 11,510 | | 13,174 | | 1,665 | | 57 | | ATG | | TAA | |
|  |  | *nad3* | | 13,175 | | 13,588 | | 414 | |  | | ATG | | TAA | |
|  |  | *atp9* | | 13,700 | | 13,924 | | 225 | | 111 | | ATG | | TAA | |
|  |  | *cox2* | | 14,046 | | 14,795 | | 750 | | 121 | | ATG | | TAA | |
|  |  | *trnR(acg)* | | 14,851 | | 14,921 | | 71 | | 55 | |  | |  | |
|  |  | *nad4L* | | 14,995 | | 15,264 | | 270 | | 73 | | ATG | | TAA | |
|  |  | *nad5* | | 15,264 | | 17,270 | | 2,007 | | -1 | | ATG | | TAA | |
|  |  | *cob* | | 17,436 | | 18,602 | | 1,167 | | 165 | | ATG | | TAA | |
|  |  | *trnC(gca)* | | 18,672 | | 18,743 | | 72 | | 69 | |  | |  | |
|  |  | *cox1* | | 19,110 | | 20,756 | | 1,647 | | 366 | | TTG | | TAA | |
|  |  | *trnR(tct)* | | 20,835 | | 20,905 | | 71 | | 78 | |  | |  | |
|  |  | *nad1* | | 21,052 | | 22,161 | | 1,110 | | 146 | | ATG | | TAA | |
|  |  | *nad4* | | 22,245 | | 23,702 | | 1,458 | | 83 | | ATG | | TAA | |
|  |  | *atp8* | | 23,774 | | 23,920 | | 147 | | 71 | | ATG | | TAA | |
|  |  | *atp6* | | 24,006 | | 24,788 | | 783 | | 85 | | ATG | | TAA | |
|  |  | *rns* | | 25,081 | | 26,396 | | 1,316 | | 292 | |  | |  | |
| *Purpureocillium xinfangense* | | *nad5* | | 3,278 | | 121,150 | | 117,873 | | 3,277 | | ATG | | TAA | |
|  |  | *cob* | | 6,099 | | 17,681 | | 11,583 | | **6,289** | | ATG | | TAG | |
|  |  | *trnC(gca)* | | 17,732 | | 17,803 | | 72 | | 2,700 | |  | |  | |
|  |  | *cox1* | | 18,159 | | 42,507 | | 24,349 | | 355 | | ATG | | TAA | |
|  |  | *trnR(tct)* | | 42,577 | | 42,647 | | 71 | | 611 | |  | |  | |
|  |  | *nad1* | | 42,844 | | 43,953 | | 1,110 | | 196 | | ATG | | TAA | |
|  | | *nad4* | | 44,050 | | 46,875 | | 2,826 | | 96 | | ATG | | TAA | |
|  |  | *atp8* | | 46,950 | | 47,096 | | 147 | | 1,124 | | ATG | | TAA | |
|  |  | *atp6* | | 47,182 | | 53,209 | | 6,028 | | 85 | | ATG | | TAA | |
|  |  | *rns* | | 53,946 | | 54,551 | | 606 | | 1,117 | |  | |  | |
|  |  | *rrnS_copy2* | | 55,853 | | 56,156 | | 304 | | 64 | |  | |  | |
|  |  | *rrnS_copy3* | | 56,949 | | 57,390 | | 442 | | 792 | |  | |  | |
|  |  | *rrnS_copy4* | | 58,519 | | 58,938 | | 420 | | 11 | |  | |  | |
|  |  | *trnY(gta)* | | 58,993 | | 59,077 | | 85 | | 54 | |  | |  | |
|  |  | *trnD* | | 59,086 | | 59,158 | | 73 | | 8 | |  | |  | |
|  |  | *trnS(gct)* | | 59,164 | | 59,246 | | 83 | | 5 | |  | |  | |
|  |  | *trnN(gtt)* | | 59,256 | | 59,326 | | 71 | | 9 | |  | |  | |
|  |  | *cox3* | | 59,369 | | 67,928 | | 8,560 | | 42 | | ATG | | TAA | |
|  |  | *trnG(tcc)* | | 67,961 | | 68,031 | | 71 | | 1,975 | |  | |  | |
|  |  | *nad6* | | 68,119 | | 70,535 | | 2,417 | | 87 | | ATG | | TAA | |
|  |  | *trnV(tac)* | | 71,930 | | 72,001 | | 72 | | 1,394 | |  | |  | |
|  |  | *trnI(gat)* | | 73,633 | | 73,704 | | 72 | | 1,631 | |  | |  | |
|  |  | *trnS(tga)* | | 73,726 | | 73,812 | | 87 | | 21 | |  | |  | |
|  |  | *trnW(tca)* | | 73,816 | | 73,887 | | 72 | | 3 | |  | |  | |
|  |  | *trnP(tgg)* | | 75,045 | | 75,116 | | 72 | | 706 | |  | |  | |
|  |  | *rnl* | | 75,177 | | 76,494 | | 1,318 | | 60 | |  | |  | |
|  |  | *rnl_copy2* | | 85,170 | | 85,241 | | 72 | | 176 | |  | |  | |
|  |  | *rnl_copy3* | | 85,638 | | 85,780 | | 143 | | 396 | |  | |  | |
|  |  | *rps3* | | 86,053 | | 87,399 | | 1,347 | | 272 | | ATG | | TAG | |
|  | | *rnl_copy4* | | 88,131 | | 88,185 | | 55 | | 731 | |  | |  | |
|  |  | *trnT(tgt)* | | 91,301 | | 91,371 | | 71 | | 602 | |  | |  | |
|  |  | *trnE* | | 92,026 | | 92,098 | | 73 | | 654 | |  | |  | |
|  |  | *trnM(cat)* | | 92,135 | | 92,205 | | 71 | | 36 | |  | |  | |
|  |  | *trnM(cat)_copy2* | | 92,768 | | 92,840 | | 73 | | 562 | |  | |  | |
|  |  | *trnL(taa)* | | 92,843 | | 92,924 | | 82 | | 2 | |  | |  | |
|  |  | *trnA(tgc)* | | 92,929 | | 92,999 | | 71 | | 4 | |  | |  | |
|  |  | *trnF(gaa)* | | 93,970 | | 94,042 | | 73 | | 369 | |  | |  | |
|  |  | *trnK(ttt)* | | 94,043 | | 94,115 | | 73 | |  | |  | |  | |
|  |  | *trnL(tag)* | | 96,752 | | 96,834 | | 83 | | 343 | |  | |  | |
|  |  | *trnQ(ttg)* | | 97,683 | | 97,756 | | 74 | | 848 | |  | |  | |
|  |  | *trnH(gtg)* | | 97,762 | | 97,836 | | 75 | | 5 | |  | |  | |
|  |  | *trnM(cat)_copy3* | | 97,862 | | 97,933 | | 72 | | 25 | |  | |  | |
|  |  | *nad2* | | 97,996 | | 106,136 | | 8,141 | | 62 | | ATG | | TAG | |
|  |  | *nad3* | | 107,400 | | 107,813 | | 414 | | 451 | | ATG | | TAA | |
|  |  | *atp9* | | 108,065 | | 109,378 | | 1,314 | | 251 | | ATG | | TAA | |
|  |  | *cox2* | | 109,509 | | 117,444 | | 7,936 | | 189 | | ATG | | TAA | |
|  |  | *trnR(acg)* | | 117,504 | | 117,574 | | 71 | | 447 | |  | |  | |
|  |  | *nad4L* | | 117,659 | | 117,958 | | 300 | | 84 | | ATG | | TAA | |

**Note:** Adjacent gene distance represents the positional relationship between two neighboring genes. Negative values indicate overlapping regions between adjacent genes, whereas positive values indicate non-overlapping intergenic regions.
